# A case report of late‐onset atypical Hemolytic Uremic Syndrome during interferon beta in multiple sclerosis: Open issues in literature review

**DOI:** 10.1002/brb3.1930

**Published:** 2020-12-16

**Authors:** Mosè Parisi, Alessia Manni, Francesca Caputo, Maria Trojano, Damiano Paolicelli

**Affiliations:** ^1^ Department of Basic Medical Sciences Neurosciences and Sense Organs University of Bari “Aldo Moro” Bari Italy

**Keywords:** adverse effects, immunomodulatory therapy, multiple sclerosis

## Abstract

**Background and aims:**

Interferon beta (IFNβ) is a well‐established first‐line therapy for relapsing–remitting multiple sclerosis (RRMS) patients and remains the most widely prescribed agent. Atypical hemolytic uremic syndrome (aHUS) represents a rare but severe adverse effect (AE) that could occur even after many years from the beginning of IFNβ therapy. Eculizumab is currently approved for treatment of aHUS and recently for neuromyelitis optica spectrum disorder (NMOSD) with aquaporin‐4 antibodies (AQP4‐IgG). In this article, we report the case of the latest onset of IFNβ‐related aHUS experienced by an MS patient and we briefly review the literature on this topic.

**Methods:**

We performed a systematic review of the literature using PubMed, and we performed a retrospective analysis of RRMS patients that received IFNβ‐1a in our center and developed thrombotic microangiopathy (TMA). From this search, we identified only one patient.

**Results:**

In the published literature, we identified 24 MS patients who received IFNβ as disease‐modifying treatment (DMT) and then developed thrombotic microangiopathy with kidney injury. The aHUS has been diagnosed in 6, all received IFNβ‐1a and the latest onset was after 15 years. We report a case of a 39‐year‐old man affected by RRMS who assumed IFNβ‐1a since 1999. In July 2018, he developed an IFNβ‐related aHUS. After the failure of plasma exchange, he underwent eculizumab, with an improvement of glomerular filtration rate and without new signs of MS activity.

**Conclusion:**

To our knowledge, this case represents the latest onset of IFNβ‐related aHUS in MS patients. Up to now, there are not literary reports about the possibility to reintroduce a DMT as add‐on therapy to eculizumab.

## Graphical abstract

Interferon‐beta (IFNβ) is a first‐line therapy for Relapsing‐Remitting Multiple Sclerosis (RRMS). Atypical Haemolytic Uremic Syndrome (aHUS) represents a rare but severe adverse effect (AE) that could occur even after many years from the beginning of IFNβ therapy. We report the case of the latest onset of IFNβ‐related aHUS experienced by an MS patient.

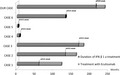

## 1 INTRODUCTION

Multiple sclerosis (MS) is chronic autoimmune disease of central nervous system (CNS) characterized by neuroinflammation, myelin, and axonal damage leading to neurodegeneration and neurological disability. Although the therapeutic landscape for MS has deeply changed in the last years, interferon beta (IFNβ) has been at the forefront of disease management strategies for more than two decades, being the first drug approved for relapsing–remitting MS (RRMS) both in the United States and Europe (Zettl et al., 2018).

IFNβ has many different immunomodulatory effects: It reduces the number of dendritic cells and downregulates antigen presentation by antigen‐presenting cells both in the peripheral blood and in the CNS (microglial cells and monocytes); it induces T regulatory cells; it decreases inflammatory T‐cell responses by inhibiting the stimulation and activation of T cells; it alters the secretion of cytokines and chemokines (Rommer et al., 2019).

Currently, IFNβ is available as IFNβ‐1a (Avonex®, Rebif®), pegylated IFNβ‐1a (Plegridy®) and IFNβ‐1b (Betaferon® or Extavia®) with preparations that differ for route (subcutaneously or intramuscular injection) and frequency of administration, which ranges from every other day/three weekly, to once a week, to biweekly (Walsh & Johnson, 2019). IFNβ‐1a most commonly reported adverse effects (AEs) are headache, flu‐like syndrome, injection‐side reaction, thyroid disorders, depression, and hepatic injury with enzyme increase. However, nephrological disorders have been described, as thrombotic microangiopathy (TMA) (Michailidou & Wilde, 2019; Walsh & Johnson, 2019), whose most frequent forms are thrombotic thrombocytopenic purpura (TTP) characterized by an insufficient ADAMTS13 activity and hemolytic uremic syndrome (HUS). Atypical HUS is an unusual form and it is unrelated to diarrhea illness. The pathogenetic mechanism underlying aHUS is not completely understood but the most accredited theory supports the association with an abnormal activation of the complement pathway (Moake, 2002). This alteration seems to be a predisposing condition which leads to endothelial dysfunction and subsequent activation, with intravascular thrombosis when precipitating factors occur (Wong et al., 2013). In the last years, some drugs have been identified as triggering factors for secondary HUS; in particular, some cases of HUS in association with IFNB are reported.

Treatment for aHUS is focused on plasma exchange and eculizumab, a humanized monoclonal antibody that prevents the cleavage of C5‐protein in C5a (anaphylatoxin effect) and C5b fragment which coordinates the formation of cell‐killing membrane attack complex (MAC) (Wong et al., 2013). Eculizumab is the first and the only drug approved for treatment of aHUS and long‐term studies confirm its efficacy and safety to improve or stabilize renal function (Menne et al., 2019). Moreover, phase III PREVENT Trial (Pittock et al., 2019) has recently demonstrated that eculizumab significantly reduced the risk of relapses compared to placebo group in patients with AQP4‐IgG‐seropositive NMOSD; this seems to be the same pathogenetic mechanism of aHUS. Currently, eculizumab is approved in EU, United States, Canada, and Japan for the treatment of AQP4‐IgG‐seropositive NMOSD (Frampton & Lana‐Peixoto, 2020).

The aim of this work was to characterize reports of TMA and aHUS for patients with MS who received subcutaneous (sc) IFNβ‐1a, to describe a case of MS patient who developed aHUS after a long time on IFNβ‐therapy and to analyze the possibility to restart a disease‐modifying treatment (DMT), after eculizumab therapy.

To our knowledge, our case represents the latest onset of aHUS on IFNβ treatment.

Moreover, in literature only 3 cases of aHUS IFNβ‐1a induced in MS patient have been treated with eculizumab, and in none of these DMT reintroduction was discussed.

## 2 METHODS

For internal validity, two researchers performed independently a systematic review of the literature using PubMed. Our search included combination of the keyword “multiple sclerosis” with “interferon beta,” “eculizumab,” “atypical Haemolytic Uremic Syndrome,” and “thrombotic microangiopathy.” Sixteen articles were found (Allinovi et al., 2017; Azkune Calle et al., 2016; Broughton et al., 2011; Etemadifar et al., 2018; Hansen et al., 2009; Hunt et al., 2014; Kimura et al., 2011; Larochelle et al., 2014; Mahe et al., 2013; Manani et al., 2017; Nerrant et al., 2013; Olea et al., 2012; Orvain et al., 2014; Rubin et al., 2014; Vosoughi & Marriott, 2014; Yam et al., 2019) and reviewed in full‐text form. A descriptive statistic is provided of the identified cases: Summaries of continuous variables have been calculated as medians with interquartile ranges (IQR) or mean and standard deviation (*SD*); categorical variables have been presented as frequencies (proportions). Moreover, in our MS Centre, we performed a retrospective analysis of RRMS patients that received IFNβ‐1a and developed TMA included on the Web platform of the Italian Multiple Sclerosis Register. From this search, we identified only one patient.

## 3 RESULTS

**Figure 1:**
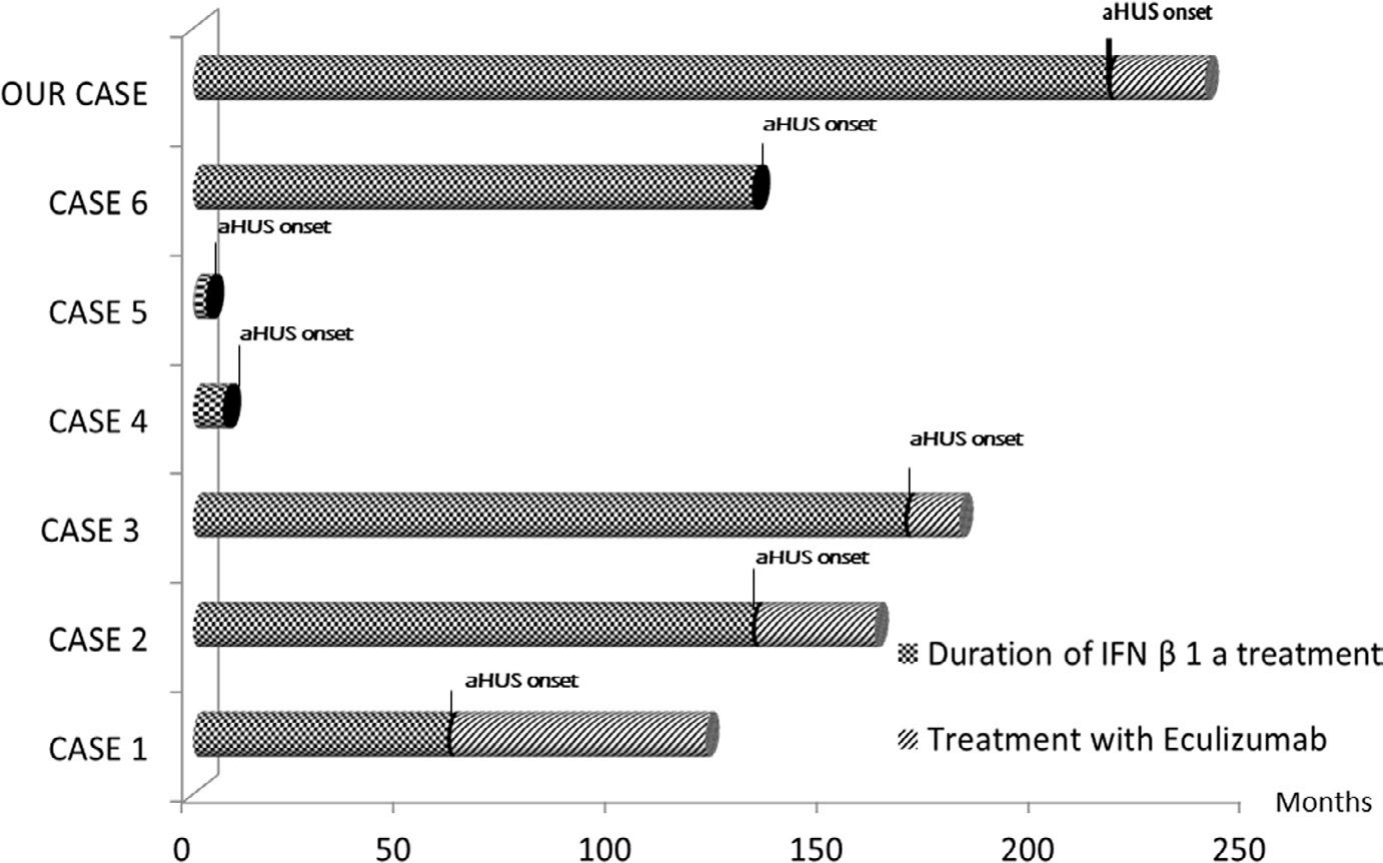
Timing of aHUS onset and duration of eculizumab treatment eventually started

In the published literature, we identified 24 MS patients who received IFNβ as DMT and then developed thrombotic microangiopathy with kidney injury (Table 1; Allinovi et al., 2017; Azkune Calle et al., 2016; Broughton et al., 2011; Etemadifar et al., 2018; Hansen et al., 2009; Hunt et al., 2014; Kimura et al., 2011; Larochelle et al., 2014; Mahe et al., 2013; Manani et al., 2017; Nerrant et al., 2013; Olea et al., 2012; Orvain et al., 2014; Rubin et al., 2014; Vosoughi & Marriott, 2014; Yam et al., 2019). In 3 of these 24 cases, the kind of INFβ used is not provided; in 4, INFβ‐1b has been used. Of the remaining 17 cases, aHUS has been diagnosed in 6, all received subcutaneous IFNβ‐1a and the latest onset was after 15 years (Figure 1); 3 of these patients received eculizumab, and in none DMT reintroduction was proposed. Of the 6 cases of aHUS, 5 were female (83%), the mean age of aHUS presentation was 39 years (range 32–47) and the onset time ranged from 3 months to 15 years with a median of 11 years. Here, we describe a clinical case of a 38‐year‐old man diagnosed according to Poser's criteria (Poser et al., 1983) with RRMS in 1998 and treated with IFNβ‐1a from 1999, at first 22 mcg, then 44 mcg. His medical and family history was unremarkable.

**Table 1:** Cases of TMA and aHUS occurring in MS patients treated with IFN‐beta reported in the literature

Author	Age in years, sex	Disease	IFNβ	Exposure to IFN‐beta	Clinical presentation	Treatment	DMT
Nerrant et al. (2013)	38,F	MS	1a	7m	aHUS, PRES	Steroids, PE	NA
Kimura et al. (2011)	36,F	MS	1a	3m	aHUS	Steroids, PE, Dialysis	NA
Allinovi et al. (2017)	46,F	MS	1a	15y	aHUS	Steroids, PE, **eculizumab**	NA
Allinovi et al. (2017)	32,F	MS	1a	11y	aHUS	PE, **eculizumab**	NA
Allinovi et al. (2017)	34,M	MS	1a	14y	aHUS	PE, **eculizumab**	NA
Manani et al. (2017)	48,F	MS	1b	15y	aHUS	Steroids, PE, **eculizumab**	NA
Larochelle et al. (2014)	47,F	MS	1a	11y	HUS	Steroids, PE	GA
Orvain et al. (2014)	52,M	MS	NA	4y	TTP	Steroids, PE, rituximab	NA
Olea et al. (2012)	37,F	MS	NA	5m	TMA	Steroids	NA
Mahe et al. (2013)	38F	MS	1a	5y	TMA	NA	NA
Hunt et al. (2014)	NA	MS	1a	8y	TMA	Dialysis	NA
Hunt et al. (2014)	NA	MS	1a	6y	TMA	Dialysis	NA
Hunt et al. (2014)	NA	MS	1a	10y	TMA	NA	NA
Hunt et al. (2014)	NA	MS	1a	6y	TMA	Dialysis	NA
Larochelle et al. (2014)	34,F	MS	1a	14m	TMA	Steroids, PE, vincristina, rituximab	NA
Larochelle et al. (2014)	41,F	MS	1a	5y	TMA, PRES	PE, Dialysis	NA
Azkune Calle et al. (2016)	36,M	MS	1a	9y	TMA	Steroids, PE	NA
Broughton et al. (2011)	53,F	MS	1b	NA	TMA	NA	NA
Etemadifar et al. (2018)	25,F	MS	1b	2y	TMA	Steroids, PE	GA
Rubin et al. (2014)	41,F	MS	NA	10y	TMA, PRES	NA	NA
Hansen et al. (2009)	41,F	MS, SLE, APS	1a	NA	TMA,	Steroids, PE, cyclophosphamide, mycophenolate	NA
Vosoughi and Marriott (2014)	41,F	MS	1a	11y	TMA, PRES	NA	NA
Vosoughi and Marriott (2014)	52,M	MS	1b	14y	TMA	PE, Dialysis	NA
Yam et al. (2019)	57,F	MS	1a	20y	TMA	NA	TF
Our case	38,M	MS	1a	18y	aHUS	PE, eculizumab	DMF

Abbreviations: aHUS, atypical hemolytic uremic syndrome; APS, antiphospholipid syndrome; F, female; GA, glatiramer acetate; M, male; m, months; MS, multiple sclerosis; NA, not available; PE, plasma exchange; SLE, systemic lupus erythematosus; TF, Teriflunomide; TMA, thrombotic microangiopathy; y, years.

INFβ therapy was well tolerated, except for flu‐like symptoms, well managed with paracetamol. He was clinically stable with an Expanded Disability Status Scale (EDSS) of 1.5. Two weeks before the admission to our hospital, he presented a history of asthenia and remitting fever; therefore, he discontinued therapy with IFNβ by himself. Then, he came to the emergency department with blurred vision, confusion and speech disorder and he showed severe hypertension, increased lactic dehydrogenase values (1,299 U/L), creatinine values (2.73 mg/dl), and decreased hemoglobin (6.5 g/dl) and platelets (57,000 cell/mm^3^). He underwent brain MRI that showed signs of vasogenic edema in parietal‐occipital lobes and brainstem like in atypical posterior reversible encephalopathy syndrome. Therefore, he was admitted to our neurological department, started strong antihypertensive therapy, and his anemia was corrected with blood cell transfusion. Microbiological and autoimmune studies on plasma and CSF, including antinuclear antibodies, were negative, just as direct Coombs test and ADAMTS13 activity. The presence of schistocytes on blood smear confirmed the hypothesis of HUS, and we suspected an atypical form for the lack of a previous history of diarrhea and the absence of fecal Shiga toxin. This hypothesis was finally supported by kidney biopsy. Visual symptoms, confusion, and radiological signs at brain MRI progressively improved and then plasma exchange (PE) was rapidly started. He underwent 9 sessions of PE, but for the persisting of severe renal failure, he started eculizumab, at a dosage of 900 mg iv weekly for 4 weeks, followed by 1,200 mg every 2 weeks. After one year and a half of follow‐up, the patient still receives eculizumab, showing stable conditions, with persistent mild CKD, well‐controlled BP, and no clinical and/or radiological MS relapses.

## 4 DISCUSSIONS

Despite the therapeutic landscape of DMTs for MS is actually wide, not all countries in the world have the same accessibility to the DMTs and IFNβ remains one of the most frequently prescribed agents with long‐term safety data. Therefore, it is important to underline also its rare AEs in real‐life settings, as aHUS, and their exact onset time, which can sometimes be longer than expected (Alonso et al., 2019; Confavreux, 1993).

Complement system represents a feature of innate immunity and is composed by circulating precursor zymogens and membrane expressed proteins that can be activated by three different ways. Complement activation has been demonstrated in both acute and chronic MS lesions and nowadays, it is well established that antibody‐ and complement‐mediated myelin phagocytosis as well as C5b‐9‐mediated lysis are involved in demyelination processes in MS and particularly in pathogenetic mechanism of NMOSD (Breij et al., 2008; Hemmer et al., 2015; Ingram et al., 2014; Lucchinetti et al., 2000; Tatomir et al., 2017).

In this setting, we may hypothesize that eculizumab could express a protective action against both renal and myelin damage, preserving the clinical and radiological course of patient's disease activity after IFNβ therapy discontinuation. The clinical case presented shows the occurrence of aHUS after 18 years of IFNβ‐1a‐treatment, and to our knowledge, it is the latest onset presented in literature; moreover, this suggests that the effect of IFNβ on the kidney function reflects a cumulative damage according to the most cases reported in literature (Mahe et al., 2013; Manani et al., 2017).

Finally, after more than one year of treatment with eculizumab, we wondered if it was necessary to reintroduce a DMT and we thought to DMF considering its benefit/risk ratio (Mills et al., 2018; Rommer et al., 2019). In particular, we have considered the safety profile of DMF and its mechanism of action: DMF seems to have protective effect against oxidative stress through the activation of nuclear factor erythroid 2‐related factor 2 (Nrf2) pathway, restoring an oxidative homeostasis. Therefore, in case of kidney injury, as aHUS, in the context of autoimmune disease like MS we can deduce that DMF could also have protective effect on the kidney from oxidative stress (Schmidlin et al., 2020). Moreover, a recently published experimental model has pointed out DMF protection potential against renal ischemia/reperfusion insult (Ragab et al., 2020). In conclusion, aHUS could occur in RRMS patients treated with IFNβ even after several years of treatment with IFNβ‐1a, suggesting how vigilance on this rare AEs can never be forgotten. Current evidence supports the efficacy of eculizumab as a first‐line therapy in patients with aHUS and treatment with eculizumab should be started as soon as possible (Zuber et al., 2012). However, up to now, there is no literary report about the possibility to reintroduce a DMT and which ones are more suitable as add‐on therapy to eculizumab.

## CONFLICT OF INTEREST

No potential conflict of interest was reported by M.P., A.M., F.C., and M.T; D.P. has served on scientific Advisory Boards for Biogen, Novartis, Roche, Merck, and Genzyme; has received speaker honoraria from Biogen Idec, Merck, Roche, Teva, Sanofi‐Genzyme, and Novartis; and has received research grants for her Institution from Biogen Idec, Merck, Roche, and Novartis. D.P. received advisory board membership, speaker's honoraria, travel support, research grants, consulting fees, or clinical trial support from Almirall, Bayer Schering, Biogen, Celgene, Excemed, Genzyme, Merck, Mylan, Novartis, Sanofi, Roche, and Teva.

## AUTHOR CONTRIBUTION

M. Parisi collected clinical data, conceived and drafted the manuscript, and created the table; A. Manni drafted the manuscript, designed the figure, and revised the literature, F. Caputo revised the literature and the manuscript in its final version, M. Trojano revised the manuscript, figure and table, D. Paolicelli supervised the work and revised the final version. All authors discussed the results and contributed to the final manuscript.

### Peer Review

The peer review history for this article is available at https://publons.com/publon/10.1002/brb3.1930.
